# Basal Autophagy and Feedback Activation of Akt Are Associated with Resistance to Metformin-Induced Inhibition of Hepatic Tumor Cell Growth

**DOI:** 10.1371/journal.pone.0130953

**Published:** 2015-06-25

**Authors:** Hua Yang, Yuan-Fei Peng, Hong-Min Ni, Yuan Li, Ying-Hong Shi, Wen-Xing Ding, Jia Fan

**Affiliations:** 1 Liver Cancer Institute, Zhongshan Hospital, Fudan University, Shanghai, 200032, China; 2 Department of Pharmacology, Toxicology and Therapeutics, The University of Kansas Medical Center, Kansas City, Kansas, 66160, United States of America; Yong Loo Lin School of Medicine, National University of Singapore, SINGAPORE

## Abstract

While accumulating evidence has shown that the use of the diabetic drug metformin may be beneficial against various tumors in some epidemiological studies, a few studies failed to show the same beneficial effects. The molecular and cellular mechanisms for these conflicting observations are not clear. In this study, we compared the inhibitory effects of cell growth by metformin on several hepatic tumor cell lines: SMMC-7721, HCC-97L, HCC-LM3 and HepG2. While metformin inhibited cell growth in all these cells, we found that SMMC-7721, HCC-97L and HCC-LM3 cells were more resistant than HepG2 cells. Mechanistically, we found that metformin inhibited mTOR in all these hepatic tumor cells. However, SMMC-7721 cells had higher levels of basal autophagy and mTORC2-mediated feedback activation of Akt than HepG2 cells, which may render SMMC-7721 cells to be more resistant to metformin-induced inhibition of cell growth. Similarly, HCC-97L and HCC-LM3 cells also had higher feedback activation of AKT than HepG2 cells, which may also account for their resistance to metformin-induced inhibition of cell growth. Therefore, the various basal autophagy and mTOR activity in different cancer cells may contribute to the controversial findings on the use of metformin in inhibition of cancers in humans.

## Introduction

Hepatocellular carcinoma (HCC) is a major cancer that accounts for more than 600,000 deaths per year [1]. HCC is very common in southeast Asia and Africa because of their high HBV infection rate. However, the incidence of HCC has increased in the US and western Europe over the past 25 years. The exact molecular pathogenesis of HCC is not yet well understood, although viral infection and alcohol abuse are responsible for the majority of HCC [2]. HCC is a highly malignant and fatal neoplasia. The survival rate in patients diagnosed at an early HCC stage is significantly improved by treatments such as surgical resection, ablation and transplantation. However, no effective treatments are available for patients with advanced or intermediate stage HCC [3].

Metformin (N, N-dimethylbiguanide) is the most widely used drug for treatment of type II diabetes [4]. Metformin lowers blood glucose levels through reduced hepatic gluconeogenesis and increased glucose update in skeletal muscles [5]. Metformin is known to activate AMP-activated protein kinases (AMPK) *in vitro* and *in vivo*, although the exact molecular mechanisms are still poorly understood [6,7]. Accumulating evidence from epidemiological studies among patients with type II diabetes who were treated with metformin suggests that metformin may lower the incidence of various cancers including liver cancer compared with those untreated [8,9,10]. However, several recent studies failed to find the beneficial effects of metformin for lowering cancer incidence in metformin users [11]. The reasons for these controversial findings are not clear.

Cancer cells can adapt to stresses or drug treatment by activating cell survival mechanism(s) such as autophagy [12]. Autophagy protects cells by providing nutrients or removing damaged organelles or toxic protein aggregates to favor cell survival. Autophagy is regulated by the cellular nutrient sensor mechanistic target of rapamycin (mTOR) and energy sensor AMPK [13]. Mammalian cells have two mTOR complexes: mTOR complex1 (mTORC1) and mTORC2, in which mTORC1 is rapamycin-sensitive but mTORC2 is rapamycin-insensitive [14]. Torin 1 is a novel and more potent mTOR inhibitor compared to rapamycin because it suppresses both mTORC1 and mTORC2 [15]. mTORC1 interacts with the ULK1-Atg13-FIP200 complex to regulate autophagy in mammalian cells [16]. Moreover, mTORC1 phosphorylates 70 kDa ribosomal protein S6 kinase (p70S6K) and the eukaryotic translation initiation factor 4E binding protein (4EBP1) to regulae protein translation. In contrast, growth factors activate mTORC2. Activated-Mtorc2 can further phosphorylate Akt to ensure the full activation of Akt, which promotes cell survival [14,17]. Under energy depletion conditions, AMPK phosphorylates TSC2 and RAPTOR, two essential regulators of mTOR, to suppress mTOR resulting in autophagy induction [18,19]. Moreover, AMPK also activates autophagy by directly phosphorylating VPS34 and Beclin 1, which are essential for autophagosome formation by providing phosphatidylinosital-3-phosphate (PI3P) [20]. Indeed, metformin is reported to increase autophagy markers in cultured cancer and normal cells as well as *in vivo* tissues [21,22]. We thus hypothesized that the lack of beneficial effects needed to lower cancer incidence in some metformin users observed in epidemiological studies could be due to alterations in autophagy and mTOR signaling.

## Materials and Methods

### Antibodies and Chemicals

Antibodies used in this study were β-actin (#A5441) from Sigma-Aldrich, p62 (#H00008878-M01) from Abnova, syntaxin 17 (#17815) from Proteintech, phosphorylated Akt (S473, #4060), Akt (#2966), phosphorylated S6 (S240/244, #5364), S6 (#2217), GAPDH (#2118) and Rab7 (#9367) from Cell Signaling Biotechnology. The secondary antibodies used in this study were HRP-conjugated goat anti-mouse (JacksonImmunoResearch, #115-035-062) or goat anti-rabbit antibodies (JacksonImmunoResearch, #111-035-045). Metformin and rapamycin were from Sigma (St. Louis, MO). The rabbit polyclonal anti-LC3B antibody was generated as described previously [23]. Chloroquine (CQ), metformin and rapamycin were from Sigma-Aldrich. All other chemicals were from Sigma, Invitrogen, or Calbiochem.

### Cell Culture

Human hepatocellular carcinoma cell line SMMC-7721 (7721), HCC97-L (97L) and HCC-LM3 (LM3) were obtained from the Liver Cancer Institute in Zhongshan Hospital (Shanghai, China) and hepatoma cell line HepG2 was from American Type Culture Collection (ATCC). 7721, 97L and LM3 were all derived from HCC patient and characterized in detail previously [24,25]. 7721, 97L, LM3 and HepG2 cells were routinely maintained in high-glucose DMEM supplemented with 10% heat-inactivated fetal bovine serum, 100 units/mL penicillin, and 100 mg/mL streptomycin. All cultures were maintained in a 37°C incubator with 5% CO_2_.

### Measurement of Cell Viability/Growth

Cell viability/growth was measured by the 3-(4, 5-dimethylthiazol-2-yl)-2, 5-diphenyltetrazolium bromide (MTT) assay or stained with Hoechst 33342 (1 μg/mL) for apoptotic nuclei or propidium iodide (PI, 1 μg/mL) for secondary necrosis or necrosis as we described previously [26]. For MTT assay, cells were seeded at a density of 5000 cells per well in 96-well plates and incubated at 37°C in a humidified 5% CO_2_ incubator for 24 hours. Serially diluted metformin was added to give the intended final concentrations. Cells were then incubated for designated time-points for up to 72 hours. Absorbance values were determined at 570 nm on a Spectra Max 250 spectrophotometer (Tecan GENios). All MTT experiments were performed in triplicate and repeated at least 3 times.

### Caspase-3 Activity Assay

This was determined as we described previously [27]. Briefly, Caspase-3 activities were measured using 30 μg of proteins and 20 μM of fluorescent substrate (Ac-DEVD-AFC, Biomol). The fluorescence signals were detected by a fluorometer (Tecan GENios) at excitation and emission wavelengths of 400 nm and 510 nm, respectively.

### Immunoblotting Analysis

Cells were washed in PBS and lysed in RIPA buffer. Thirty micrograms of protein from each sample were separated by SDS-PAGE and transferred to PVDF membranes. The membranes were stained with primary antibodies followed by secondary horseradish peroxidase-conjugated antibodies. The membranes were further developed with SuperSignal West Pico chemiluminescent substrate (Thermo Fisher).

### Adenovirus-RFP-GFP-LC3 Infection and Fluorescence Microscopy

RFP-GFP-LC3 plasmid was subcloned into an adenovirus shuttle vector. The adenovrius-RFP-GFP-LC3 was amplified in HEK293 cells and purified using CsCl. To examine autophagy, cells were seeded in a 12 well-plate (2 × 10^5^ in each well) and infected with adenovirus-RFP-GFP-LC3 (10 MOI) overnight. Cells were either untreated (control) or treated with metformin (10 mM) for 24 hours. After treatment, cells were fixed with 4% paraformaldehyde in phosphate buffered saline (PBS) and kept at 4°C for fluorescence microscopy. Fluorescence images were acquired under a Nikon Eclipse 200 fluorescence microscope with MetaMorph software.

### Electron Microscopy

Cells were either untreated (control) or treated with metformin (10 mM) for 24 hours. After treatment, cells were fixed with 2.5% glutaraldehyde in 0.2 M sodium cacodylate buffer (pH 7.4), followed by 1% OsO_4_. After dehydration, thin sections were stained with uranyl acetate and lead citrate for observation under a JEM 1011CX electron microscope (JEOL). Images were acquired digitally. The average number of autophagosomes and autolysosomes from each cell was determined from a randomly selected pool of 15 to 20 fields under each condition.

### Statistical Analysis

Experimental data were subjected to Student t-test or One-way analysis of variance analysis (ANOVA) where appropriate. p<0.05 was considered significant.

## Results

### Metformin Differentially Inhibits Cell Growth of SMMC-7721 and HeG2 Cells

**Fig 1:**
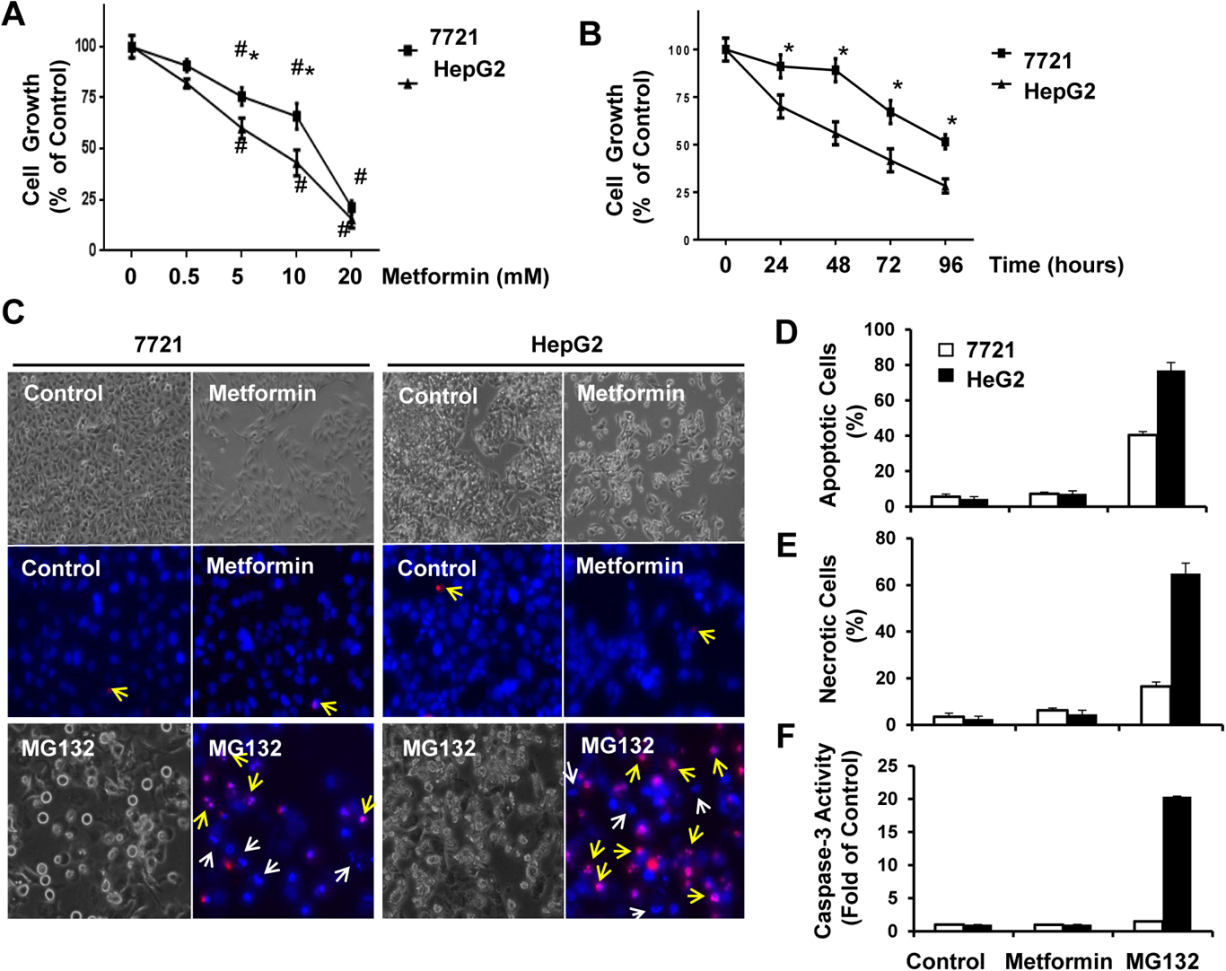
Metformin differentially inhibits cell growth of SMMC-7721 and HepG2 Cells. **(A-B)** 7721 and HepG2 cells were treated with metformin for different time points up to 96 hours or different concentrations of metformin for 72 hours followed by MTT assay. Data are presented as means ± SE (n = 3). *: p<0.05, 7721 vs HepG2 cells (Student t test). #: p<0.05, metformin vs control (One-way ANOVA analysis). (**C**) 7721 and HepG2 cells were either non-treated or treated with metformin for 72 hours or MG132 (1 μM) for 24 hours followed by phase-contrast microscopy or stained with Hoechst 33342 (1 μg/mL) and PI (1 μg/mL) followed by fluorescence microscopy. Representative phase-contrast and fluorescence photographs were shown. White arrows denote apoptotic nuclei; yellow arrows denote PI positive secondary necrotic nuclei. (**D-E**) The apoptotic and PI positive nuclei were counted from at least 3 random images. Data are presented as means ± SE (n = 3). (**F**) Total cell lysates were used to determine the caspase-3 activity using a specific fluorescence substrate. Data are presented as means ± SE (n = 3).

SMMC-7721 (7721) and HepG2 cells were treated with metformin using various concentrations and time points for up to 96 hours. We then determined cell growth using MTT assay. We found that metformin significantly decreased cell growth in a dose- and time-dependent manner. Intriguingly, 7721 cells were more resistant to metformin-induced cell growth inhibition compared to HepG2 cells (**Fig 1A & 1B**). Since the MTT assay mainly correlates with mitochondrial NAD(P)H-dependent oxidoreductase activity, reduced values in the MTT assay could be either due to decreased cell number (inhibition of cell growth) or increased cell death. To further determine whether metformin inhibited cell growth or induced cell death, we determined cell death after metformin treatment by a12ssessing the apoptotic nuclei using Hoechst 33342 nuclear staining and propidium iodide (PI) staining for necrosis or secondary necrosis. We also assessed caspase-3 activity using fluorometric analysis as we described previously [28]. We did not find any significant fragmented/condensed apoptotic nuclei or increased PI positive cells (**Fig 1C & 1E**), and caspase-3 activity was also not increased (**Fig 1F**). However, MG132, a proteasome inhibitor, markedly increased the number of apoptotic and PI positive secondary necrotic cells as well as caspase-3 activity in both 7721 and HepG2 cells although HepG2 cells were also more sensitive than 7721 cells in response to MG132. These results suggest that the reduced MTT values after metformin treatment in 7721 and HepG2 cells were mainly due to inhibition of cell growth. **Fig 1C** clearly showed reduced cell numbers in metformin-treated 7721 and HepG2 cells compared to their control un-treated cells based on the phase-contrast images. Moreover, the reduced cell growth was more dramatic in metformin-treated HepG2 cells than in 7721 cells, which is consistent with the MTT assay. Taken together, these results suggest that metformin differentially inhibits 7721 and HepG2 cell growth but not cell death, and HepG2 cells are more sensitive to metformin-induced inhibition of cell growth than 7721 cells.

### Feedback Activation of Akt after Metformin Treatment May Contribute to the Resistance of Metformin-Induced Cell Growth Inhibition

**Fig 2:**
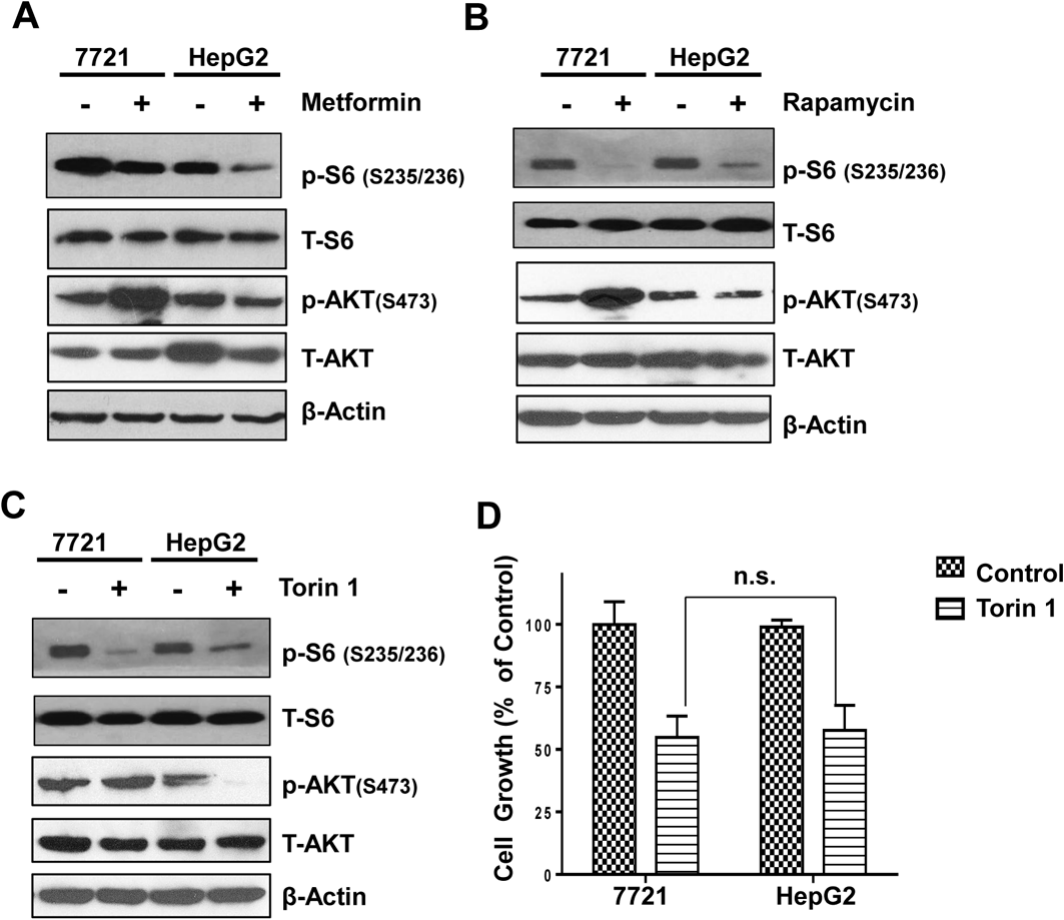
Changes of mTOR and Akt after metformin treatment in 7721 and HepG2 cells. (**A-C**) 7721 and HepG2 cells were treated with metformin (10 mM), rapamycin (10 μM) or Torin 1 (200 nM) for 24 hours. Total cell lysates were subjected to immunoblot analysis and representative blots from 3 independent experiments are shown. (**D**) 7721 and HepG2 cells were treated with Torin 1 (200 nM) for 72 hours followed by MTT assay. Data are presented as means ± SE (n = 3). ns: no significant statistical difference, 7721 vs HepG2 cells (Student t test).

We next determined the activation of mTOR and Akt after metformin treatment in 7721 and HepG2 cells. The phosphorylated level of S6, a substrate protein of mTOR, is often increased when mTORC1 activity is increased. We found that metformin treatment decreased the phosphorylated levels of S6 in both 7721 and HepG2 cells (**Fig 2A**), suggesting that metformin inhibits mTROC1 in both 7721 and HepG2 cells. Akt is a key regulator of cell survival that is phosphorylated at threonine (T308) for activation by the upstream kinase PDK1 in response to growth factors. However, phosphorylation of Akt at a serine residue (S473) by mTORC2 is required for the full activation of Akt. Intriguingly, we found that treatment with metformin increased the phosphorylated levels of Akt (S473) only in 7721 but not in HepG2 cells (**Fig 2A**). Rapamycin, a known mTORC1 inhibitor, also increased the phosphorylated levels of Akt (S473) in 7721 but not in HepG2 cells (**Fig 2B**). In contrast, Torin 1, a potent inhibitor for both mTORC1 and mTORC2, did not further increase the phosphorylated levels of Akt (S473) in 7721 cells (**Fig 2C**). These data suggest that the increased phosphorylated levels of Akt (S473) in metformin-treated 7721 cells were likely due to increased mTORC2 activity. Interestingly, unlike metformin, the inhibitory effects on cell growth induced by Torin 1 were almost identical in 7721 and HepG2 cells (**Fig 2D**). These results suggest that the differential inhibitory effects on cell growth induced by metformin in 7721 and HepG2 cells were likely partially due to the activation of mTORC2 by metformin in 7721 cells, which resulted in increased Akt activation to favor cell survival.

### 7721 Cells Have Higher Basal Autophagy than HepG2 Cells Regardless of Metformin Treatment

**Fig 3:**
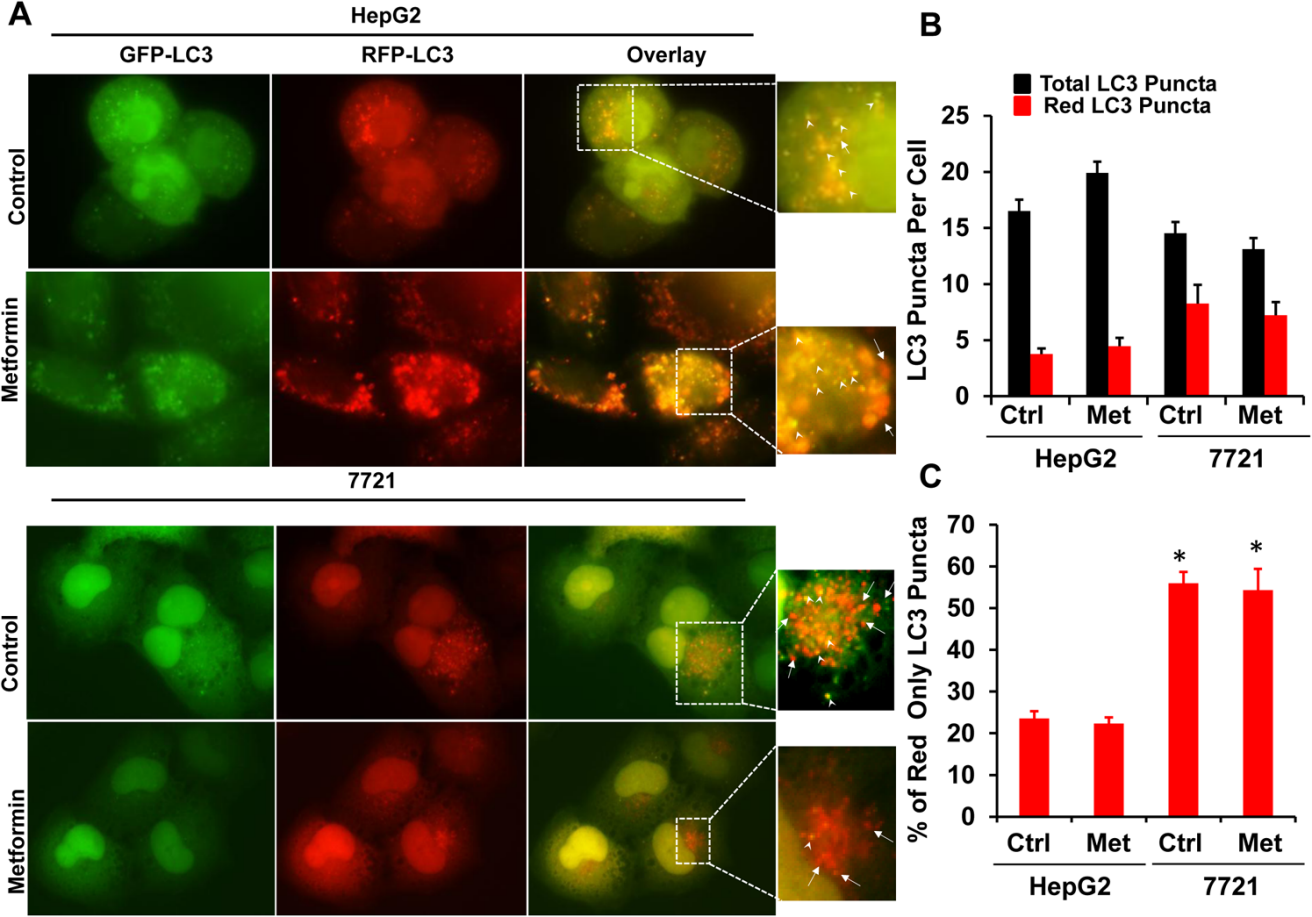
7721 cells have higher basal autophagy than HepG2 cells regardless of metformin treatment. **(A).** 7721 and HepG2 cells were infected with adenovirus RFP-GFP-LC3 (10 MOI) overnight and further treated with metformin (10 mM) for another 24 hours followed by fluorescence microscopy. Representative GFP-LC3, RFP-LC3 and overlay images are shown. Right panels are enlarged photographs from the boxed areas. Arrow heads: yellow puncta; arrow heads: red only puncta. (**B-C)** The number of yellow and RFP-LC3 (red only) puncta per cell was quantified. Total number of LC3 puncta is the sum of the number of yellow puncta and red only puncta. Data are presented as means ± SE (n = 3 independent experiments). * p<0.05, 7721 vs HepG2 (Student t test).

**Fig 4:**
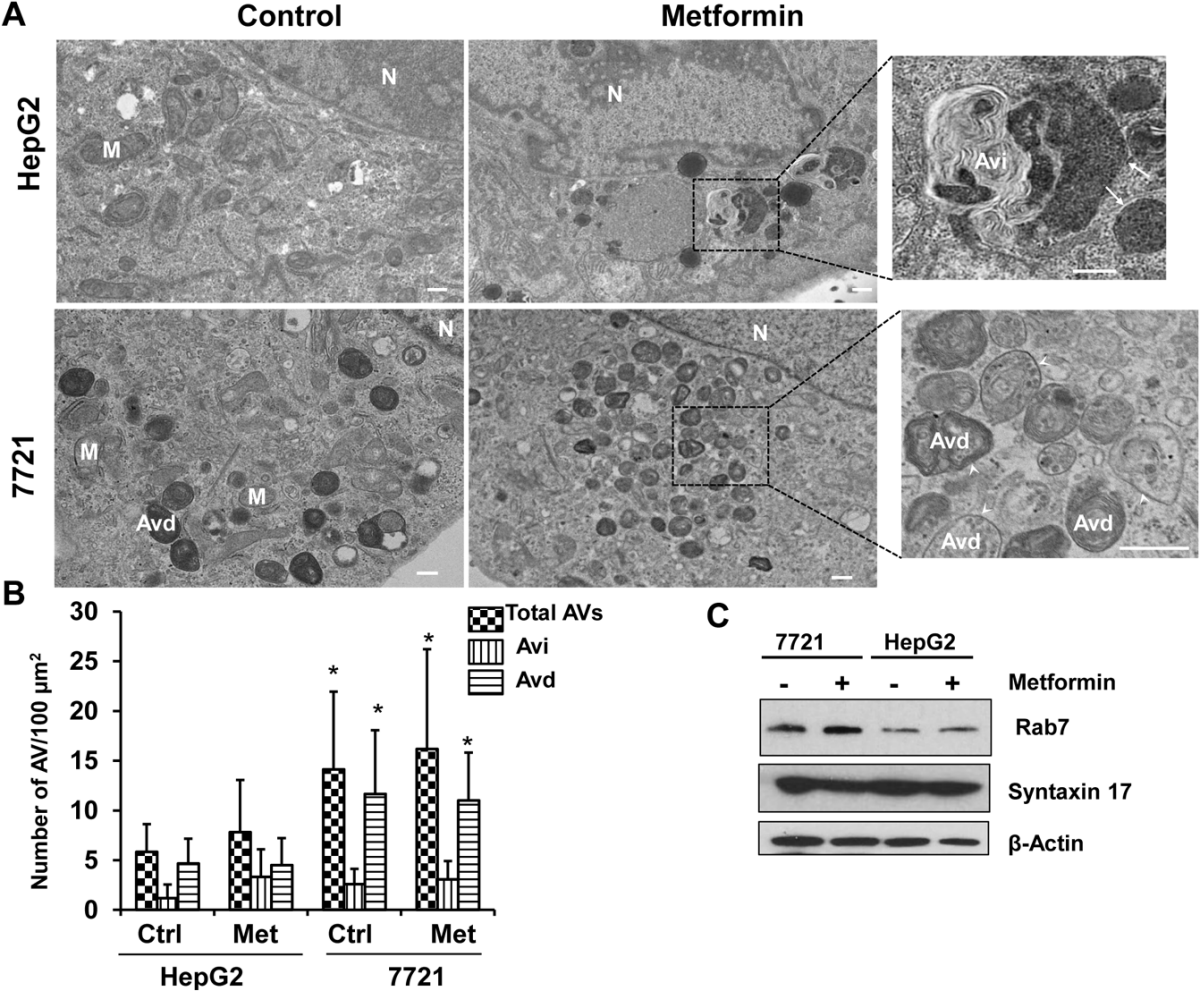
7721 cells have higher basal autophagy than HepG2 cells likely due to increased fusion of autophagosomes with lysosomes. 7721 and HepG2 cells were treated with metformin (10 mM) for 24 hours followed by EM studies. Representative EM images are shown in (**A**). Right panels are enlarged EM photographs from the boxed areas. M: Mitochondria; N: Nuclei. Bar: 500 nm. Arrows: Avi, arrow heads: Avd. (**B**) The number of autophagic vacuoles (AVs, including both Avi and Avd), Avi and Avd per 100 μm^2^ cytosol was quantified. Data are presented as means ± SE (more than 15 cell sections). * p<0.05, 7721 vs HepG2 (Student t test). (**C**) 7721 and HepG2 cells were treated with metformin for 24 hours. Total cell lysates were subjected to immunoblot analysis. Representative blots are shown from three independent experiments.

To determine autophagy in metformin-treated 7721 and HepG2 cells, we infected 7721 and HepG2 cells with an adenovirus RFP-GFP-LC3 for 24 hours followed by metformin treatment. It is known that when RFP-GFP-LC3 labeled autophagosomes fuse with lysosomes, the GFP signals are quenched due to the acidic environment in the autolysosomes, but the RFP signals are relatively stable [29]. Therefore, an increased number of RFP-LC3 (red only) puncta can be used to reflect autophagic flux. There was no difference for the total number of LC3 puncta (yellow plus red only puncta) among HepG2 and 7721 cells regardless of metformin treatment (**Fig 3A & 3B**). However, the number of RFP-LC3 puncta (red only) was significantly higher in control (un-treated) 7721 cells than in control (un-treated) HepG2 cells, suggesting that there was significantly higher basal autophagy in 7721 cells compared to HepG2 cells. Intriguingly, further treatment with metformin did not change either the total or red only LC3 puncta numbers in both 7721 and HepG2 cells (**Fig 3C**). EM studies also revealed that there was an increased total number of autophagosomes (early autophagosomes (Avi) plus late autolysosomes (Avd)) in 7721 cells than HepG2 cells. Interestingly, the number of Avd (single membrane vesicles with degrading electron dense contents) (**Fig 4A, arrow heads**) but not Avi (double membrane vesicles) (**Fig 4A, arrows**) was significantly higher in 7721 cells compared to HepG2 cells. Similar to the observations from the RFP-GFP-LC3 assay, metformin treatment did not alter the number of total AV or AVd in either 7721 or HepG2 cells (**Fig 4B**). The fusion of autophagosomes with lysosomes is mediated by the small GTPase Rab7 and an autophagosomal SNARE protein syntaxin 17. We found that 7721 cells had much higher basal protein levels of Rab7, but not syntaxin 17, compared to HepG2 cells (**Fig 4C**). Collectively these data suggest that 7721 cells have higher basal autophagy and likely higher efficiency for the fusion of autophagosomes with lysosomes.

### Metformin Fails to Induce Autophagic Flux in 7721 and HepG2 Cells in Prolonged Culture Conditions

**Fig 5:**
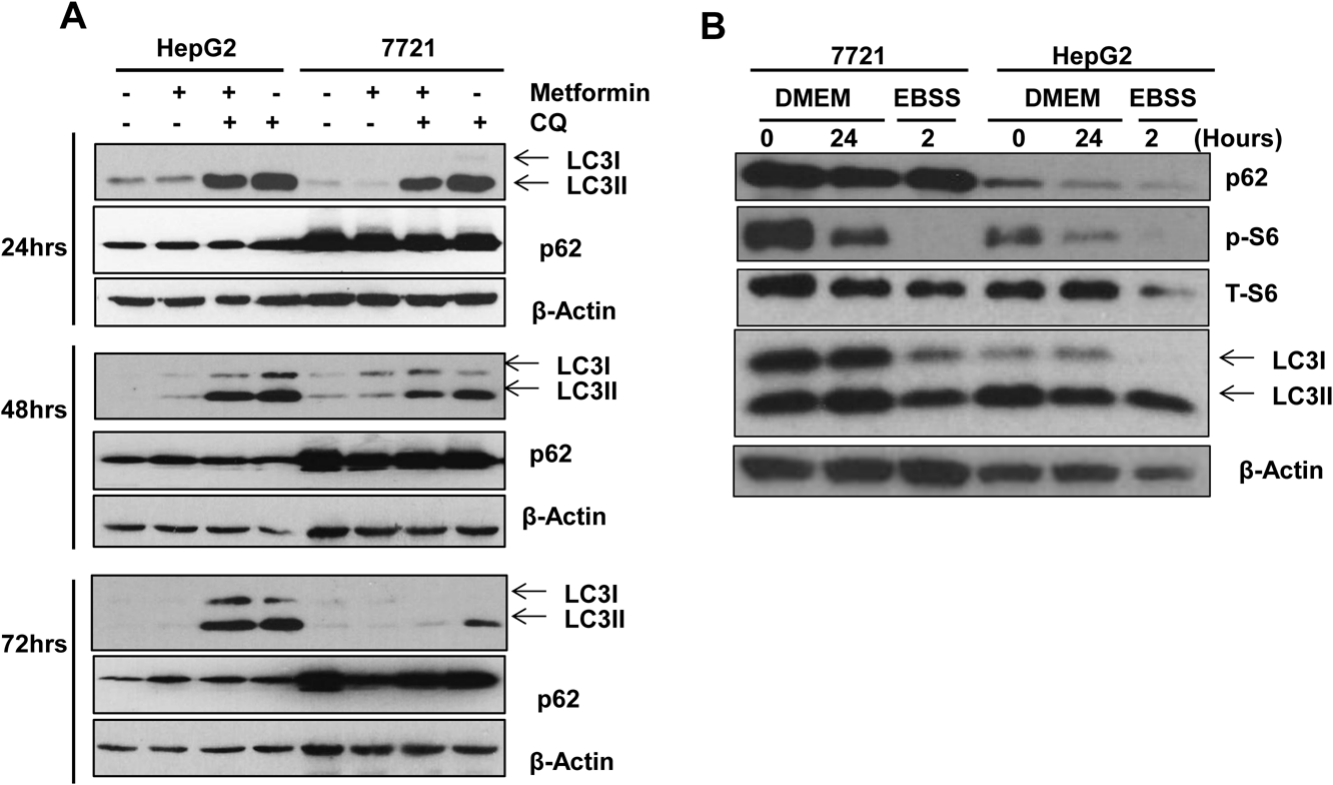
Metformin does not induce autophagic flux in 7721 and HepG2 cells during prolonged culture conditions. **(A)** 7721 and HepG2 cells were treated with metformin (10 mM) in the presence or absence of CQ (20 μM) for 24, 48 and 72 hours. CQ was added at the last four hours before the cells were harvested. Total cell lysates were subjected to immunoblot analysis. Representative blots are shown from three independent experiments. (**B**) 7721 and HepG2 cells were cultured in full DMEM medium for 0 (after cells were trypsinized and attached overnight) or 24 hours (culture for 24 hours after overnight attachment) or cultured in EBSS buffer for 2 hours. Total cell lysates were subjected to immunoblot analysis. Representative blots are shown from three independent experiments.

We next determined autophagic flux in metformin-treated 7721 and HepG2 cells in the presence and absence of the lysosomal inhibitor chloroquine (CQ). As shown, metformin alone treatment barely changed the levels of LC3-II compared to the un-treated control cells in both 7721 and HepG2 cells after either 24, 48 or 72 hours treatment. While CQ treatment alone increased the levels of LC3-II in all the time points that we assessed, the levels of LC3-II did not further increase in cells that were treated with CQ plus metformin in either 7721 or HepG2 cells (**Fig 5A**). These data suggest that metformin did not induce autophagic flux in either 7721 or HepG2 cells, which was consistent with the results from the RFP-GFP-LC3 puncta assay and EM studies even though metformin inhibits mTOR in both 7721 and HepG2 cells. Metformin treatment also did not affect the levels of p62/SQSTM1 in both 7721 and HepG2 cells, a protein that is normally degraded during starvation-induced autophagy, but the basal level of p62 in 7721 cells was much higher than that of HepG2 cells. It is possible that the high basal level of autophagy in 7721 and HepG2 cells may offset the effects of metformin on autophagy activation. To test this hypothesis, we determined the changes of mTOR and basal autophagy under our culture conditions without metformin treatment. We found that there was a decrease in the phosphorylated S6 and p62 levels, although the levels of LC3-II were less affected in both 7721 and HepG2 cells after 24 hours culture in full culture medium. As a positive control for autophagy, we cultured 7721 and HepG2 cells in nutrient free EBSS buffer for 2 hours. We found that the levels of phosphorylated S6 and p62 were also decreased (**Fig 5B**). These data suggest that 7721 and HepG2 cells increase autophagic flux during the culture conditions even under nutrient enriched medium.

### Pharmacological Inhibition of Autophagy Sensitizes HCC Cells to Metformin-Induced Inhibition of Cell Growth

**Fig 6:**
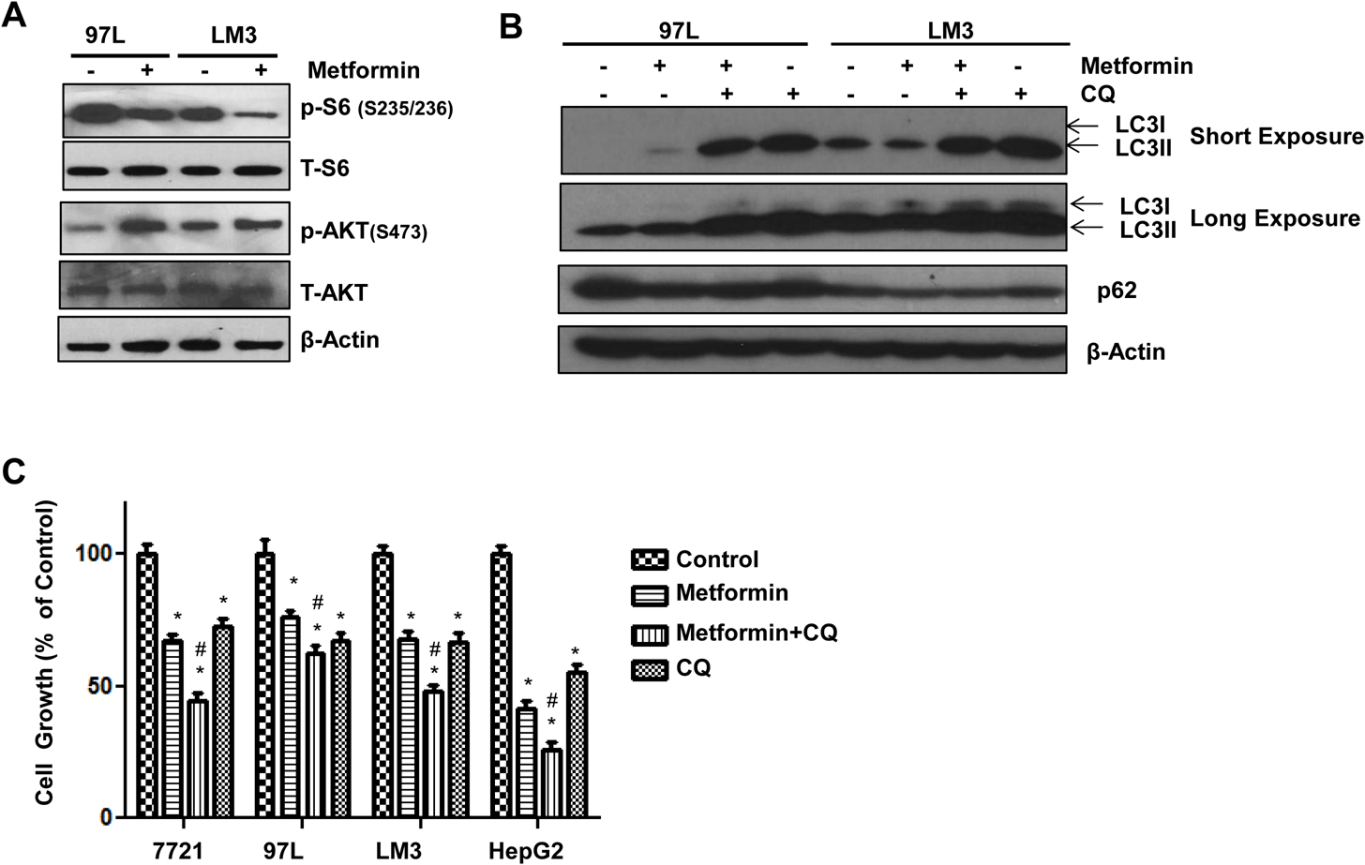
Pharmacological Suppression of autophagy sensitizes 7721, 97L, LM3 and HepG2 cells to metformin-induced inhibition of cell growth. (**A**) 97L and LM3 cells were treated with metformin (10 mM) for 24 hours. Total cell lysates were subjected to immunoblot analysis and representative blots from 3 independent experiments are shown. (**B**) 97L and LM3 cells were treated with metformin (10 mM) in the presence or absence of CQ (20 μM) for 24 hours. Total cell lysates were subjected to immunoblot analysis. Representative blots are shown from three independent experiments. (**C**) 7721, 97L, LM3 and HepG2 cells were treated as in (**B**) for 72 hours followed by MTT assay. Data are presented as means ± SE (n = 3). *: p<0.05, treatment vs control, # p<0.05 metformin vs metformin+CQ (One-way ANOVA analysis).

To determine whether the feedback activation of Akt and lack of autophagic flux that we observed in 7721 cells after metformin treatment would also occur in other HCC cells, 97L and LM3, two other HCC cells were treated with metformin. Similar to 7721 cells, we found that metformin treatment decreased the levels of phosphorylated S6 but increased the phosphorylated levels of Akt (S473) in 97L and LM3 cells (**Fig 6A**). Metformin treatment also failed to induce autophagic flux in 97L and LM3 cells (**Fig 6B**), which was also consistent with the results that we observed in 7721 cells. These results suggest that 97L and LM3 cells may behave similar to 7721 cells but are different from HepG2 cells. Indeed, results from MTT assay revealed that 97L and LM3 cells were also resistant to metformin-induced growth inhibition compared to HepG2 cells. When all these four different HCC cells were treated with metformin in the presence of CQ, CQ treatment further reduced the cell growth compared to metformin alone (**Fig 6C**). These data suggest that inhibition of autophagy may further sensitize 7721, 97L, LM3 and HepG2 cells to metformin-induced cell growth inhibition even though metformin did not induce autophagic flux in these cells, which was likely due to high basal autophagy levels in these cells. Taken together, our results suggest that the basal autophagy level and feedback activation of Akt in different HCC cells may account for their different sensitivities in response to metformin.

## Discussion

mTOR and Akt are the two key enzymes that regulate cell growth. Activation of mTOR increases cellular protein and lipid synthesis, which are essential building blocks for cell growth. Akt is often highly activated in cancer cells and phosphorylates numerous cellular substrates to favor cell survival and growth. In addition, mTOR and Akt can also regulate each other. In the presence of nutrients and growth factors, Akt is activated and further activates downstream mTOR. On the other hand, activated mTORC2 can also phosphorylate Akt (S473) to ensure the full activation of Akt [14,17]. While some epidemiological studies suggest that the use of metformin may have a beneficial effect against cancer including liver cancer, some other studies failed to find any correlation between the use of metformin and cancer prevention [8,9,10,11]. The reasons for these inconsistent results are not clear. Our results from this study suggest that metformin may suppress cancer cell growth via inhibition of mTOR, but the differential basal autophagy and feedback activation of Akt induced by metformin in certain cancer cells may contribute to resistance to metformin-induced inhibition of cell growth.

It is generally thought that autophagy serves as a cell survival mechanism resulting in the resistance of cancer cells to many stressors including traditional chemotherapy treatments. Although metformin treatment failed to further enhance autophagic flux in either 7721 or HepG2 cells, the basal level of autophagy was much higher in 7721 cells than in HepG2 cells, which could partially explain why 7721 cells were resistant to metformin-induced inhibition of cell growth compared to HepG2 cells. Moreover, 7721 cells also had higher phosphorylated Akt (S473) than HepG2 cells after metformin treatment, likely due to the mTORC2 activation in 7721 cells, which can also favor the survival of 7721 cells compared to HepG2 cells. Indeed, in addition to 7721 cells, 97L and LM3, two other HCC cell lines, also had higher feedback activation of Akt after metformin treatment and were also resistant to metformin-induced growth inhibition. The mechanisms by which 7721, 97L and LM3 cells have higher mTORC2-mediated Akt activation than HepG2 cells after metformin treatment are not clear. Future studies may determine whether there are any differences for mTORC2 components in 7721, 97L, LM3 and HepG2 cells.

Autophagy is negatively regulated by mTOR, the key sensor of cellular nutrients. In contrast, autophagy is positively regulated by AMPK, the key sensor of cellular energy, by at least three mechanisms. Firstly, AMPK phosphorylates TSC2 and RAPTOR, two essential regulators of mTOR, to suppress mTOR. Secondly, AMPK directly phosphorylates VPS34 and Beclin 1 to activate the VPS34-Beclin 1 complex, which is essential for autophagosome formation by providing phosphatidylinosital-3-phosphate (PI3P) [20]. Thirdly, AMPK can also directly phosphorylate ULK1 and activate ULK1 to activate autophagy.

Perhaps one of the most intriguing findings in this study was that metformin did not further increase autophagic flux in 7721, 97L, LM3 and HepG2 cells even though metformin inhibited mTOR in all these cell lines. However, 7721 and HepG2 cells already had high levels of basal autophagy during culture, which may prevent further induction of autophagy by metformin treatment. Nevertheless, autophagy normally promotes growth of cancer cells because pharmacological inhibition of autophagy enhanced the inhibitory effects of metformin on the growth of 7721, 97L, LM3 and HepG2 cells.

In conclusion, we found that the diabetic drug metformin can inhibit hepatic tumor cell growth *in vitro* likely through the inhibition of mTOR. However, different levels of basal autophagy and feedback activation of Akt by mTORC2 in different hepatic tumor cells may determine the sensitivity of these cells in response to metformin-induced growth inhibition. Future works are definitely needed to further determine whether the basal autophagy and Akt activation may contribute to the controversial findings on the use of metformin in inhibition of cancers in humans by studying animal models or directly assessing human cancer samples.
